# Deuterium Concentration as a Dual Regulator: Depletion and Enrichment Elicit Divergent Transcriptional Responses in A549 Lung Adenocarcinoma Cells

**DOI:** 10.3390/ijms27062605

**Published:** 2026-03-12

**Authors:** Gábor I. Csonka, Ildikó Somlyai, Gábor Somlyai

**Affiliations:** 1 Department of Physics and Engineering Physics, Tulane University New Orleans, New Orleans, LA 70118, USA; 2 HYD LLC for Cancer Research and Drug Development, 1119 Budapest, Hungary; isomlyai@hyd.hu (I.S.); gsomlyai@hyd.hu (G.S.)

**Keywords:** deuterium modulation, A549 cells, NanoString profiling, drug resistance, growth factor signaling, transcriptional amplification, lung adenocarcinoma

## Abstract

Deuterium abundance has been proposed as a modulator of cellular metabolism; however, its influence on cancer-associated gene expression networks remains incompletely characterized. We analyzed A549 lung adenocarcinoma cells cultured across four deuterium concentrations (40, 80, 150, and 300 ppm) using NanoString nCounter profiling. Expression data were processed through multistep filtering, symbolic trajectory encoding, and density-based spatial clustering (DBSCAN) to identify extreme expression responders, and Gaussian mixture modeling (GMM-6) to resolve coordinated gene-expression modules. DBSCAN identified 11 outlier genes under deuterium depletion, including reduced expression of multidrug-resistance–associated *ABCB1* (−42% at 80 ppm), proliferative signaling component *FGFR4* (−19%), and transcriptional amplifier *MYCN* (−24%). In contrast, enrichment at 300 ppm produced a broad increase in oncogenic expression (mean +44%), with marked elevation of inflammation-related (*IL6*, *TGFBR2*) and invasion-associated (*MMP9*) genes. GMM-6 clustering of the remaining core network resolved six functional modules, indicating that depletion preferentially reduces expression of genes associated with plasticity-related programs (Cluster 5: *TGFB1*, *S100A4*), while basal survival-associated genes (Cluster 6: *BIRC5*, *RET*) remain comparatively stable. Together, these results indicate that deuterium concentration acts as a bidirectional modulator of gene expression programs in the A549 model, with enrichment broadly elevating oncogenic expression and moderate depletion associated with selective downregulation of genes linked to resistance, signaling, and invasive behavior. Significance: Deuterium depletion is associated with reduced expression of genes involved in multidrug resistance, growth-factor signaling, and transcriptional amplification, revealing deuterium-responsive transcriptional vulnerabilities within the A549 lung adenocarcinoma model.

## 1. Introduction

Understanding the molecular architecture of oncogenic progression is a central challenge in cancer biology. Transcriptional programs shape the molecular processes underlying the Hallmarks of Cancer, enabling non-mutational epigenetic reprogramming and deregulated cellular energetics. While conventional tumor promoters and suppressors foster clonal expansion through established signaling cascades, emerging evidence indicates that modulation of deuterium concentration—particularly depletion— may represent a distinct, yet underexplored, potential regulatory layer. A shift in deuterium concentration from standard 150 ppm to 80 ppm has been shown experimentally to influence mitochondrial function and redox balance in A549 lung adenocarcinoma (LUAD) [1]. This exerts downstream effects on sustaining proliferative signaling and drug sensitivity [1], and signal transduction [2], with potential consequences for cell-cycle progression, the emergence of drug resistance [3], and the selective metabolic targeting of malignant cells, influencing their capacity for deregulating cellular energetics [4].

The role of deuterium as a regulator of cellular physiology was first proposed in the early 1990s, when Somlyai et al. demonstrated that reducing naturally occurring deuterium content interferes with tumor cell growth [5]. Subsequent studies extended these findings by reporting that deuterium-depleted water (DDW) reduces the expression of cancer-related genes, including c-myc, Ha-ras and p53, in carcinogen-treated mice [6], as well as Kras, Bcl2, and Myc in lung cancer models [7].

These observations raise the question of whether such gene-level effects extend to human cancer models. To answer this question, Cong et al. [8] demonstrated that 50 ± 5 ppm DDW inhibited A549 LUAD cell growth and induced apoptosis in vitro (effectively overcoming the cells’ mechanisms for resisting cell death, supported by electron microscopy and flow-cytometric analysis) and reduced tumor growth in a mouse xenograft model in vivo. However, despite these phenotypic observations, the broader transcriptomic landscape underlying these metabolic and survival effects is insufficiently characterized. Therefore, the present study addresses this gap by identifying the specific gene expression markers that mediate these experimentally validated metabolic and apoptotic phenotypes.

Kovács et al. [9] conducted a comprehensive in vitro study using the A549 LUAD cells to examine how altered deuterium concentrations affect gene expression. Prior screening had shown that A549 cells are particularly sensitive to deuterium modulation compared to other cell lines [1], supporting their use as a representative model. Cells were cultured in media containing 40, 80, 150 (control), and 300 ppm deuterium, and expression output of technical duplicates was quantified with NanoString technology [10] across 236 cancer-related and 536 kinase genes. Expression changes were evaluated under permissive thresholds (≥30% deviation from control; ≥30 mRNA copies), given the noise level in the results. The study revealed that 97.3% of cancer-related genes were upregulated at 300 ppm, whereas only five genes were downregulated under depletion, highlighting strong activation under enrichment and modest suppression under depletion.

In our previous work [11], we reanalyzed the same A549 dataset with a focus on data quality. Recognizing that raw NanoString counts are prone to stochastic noise, especially at low expression levels, we exploited technical duplicates to calculate the coefficient of variation (CV) for each gene, thereby quantifying measurement precision. Genes with high CVs or low (<20) counts were excluded, ensuring that subsequent conclusions were drawn only from reliable, high-confidence signals. This refined analysis revealed that deuterium concentration not only drives broad increases in oncogenic gene expression but also specifically modulates networks governing drug resistance, survival, and phenotypic plasticity across nine distinct expression patterns. These effects were obscured in the noisier raw dataset.

In our current work, we build on this foundation but adopt a different filtering strategy. The rationale for choosing 40, 80, 150, and 300 ppm deuterium concentration is grounded in prior experimental evidence demonstrating that deuterium concentration directly influences cancer cell proliferation and metabolic signaling, as detailed in Section 4.1. Rather than relying on CV and robust Z-score thresholds, we calculated propagated relative errors across the three non-control concentrations (40, 80, and 300 ppm) and retained only genes with a summed relative error < 0.55. This threshold selection was empirically guided by the distribution of error values in this dataset to include core tumor suppressors, major oncogenes, cancer-related apoptosis and survival regulators, transcription factors with strong cancer roles, and invasion, metastasis, and EMT-associated genes. The last gene that satisfies this criterion is *PTGS2*, a major tumor-promoting gene, heavily involved in inflammation-driven tumor progression, and proven to be a key gene in this analysis. As a result, 102 genes were included compared to 87 in our previous study, with 37 biologically relevant additions, such as *TP53*, *NRAS*, *IL6*, and *PTEN*, and 22 removals due to excessive propagated error. This refinement yields a gene set that is both more accurate and biologically comprehensive, providing a stronger basis for downstream clustering and pathway analysis.

This study employed the A549 cell line, a representative model of *KRAS*-driven LUAD characterized by co-occurring *KRAS* G12S, *STK11* (*LKB1*) loss, and *KEAP1* G333C mutations on a wild-type *TP53* background [12,13]. These alterations are high-risk genetic mutations in which *KRAS* activation and *STK11* deficiency cooperate to promote proliferative signaling and metabolic deregulation, while *KEAP1* inactivation reinforces oxidative-stress resilience through constitutive NRF2 activation. The retention of wild-type *TP53* preserves DNA-damage signaling competence, positioning A549 as a robust model for dissecting how *KRAS*-mutant LUAD integrates metabolic adaptation, drug resistance programs, and stress-response circuitry. This supports the use of A549 as a platform for mapping deuterium-linked regulation in aggressive, *KRAS*-driven disease. Thus, our results specifically characterize the *KRAS/STK11/KEAP1* co-mutant LUAD subtype and should not be generalized to genetically distinct forms of LUAD.

The present study introduces a refined analytical pipeline that integrates symbolic clustering, trajectory mapping, and rigorous data cleaning. Using density-based spatial clustering (DBSCAN) to detect sentinel outliers and Gaussian mixture modeling (GMM-6) to resolve coherent gene expression modules, this framework advances prior work [9,11] by resolving the gene regulatory networks governing metabolic plasticity and defining the specific gene expression signatures associated with invasive and drug-resistant phenotypes.

## 2. Results

### 2.1. The Oncogenic Core Network

**Figure 1:**
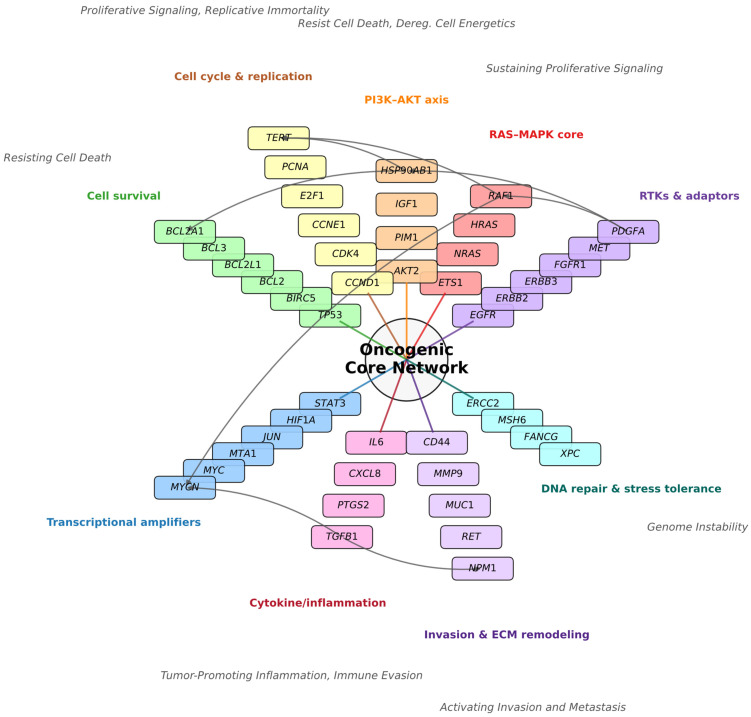
Oncogenic core network architecture of selected A549 lung adenocarcinoma genes. The schematic illustrates the functional organization of 102 consensus cancer genes into nine interconnected color coded pathways. Nodes represent functional modules (e.g., *RAS–MAPK* core, *PI3K–AKT* axis), while edges depict canonical signaling dependencies. Upstream receptor tyrosine kinases (e.g., *EGFR*, *MET*) transmit growth signals to proliferative and survival cascades; Cytokine signaling integrates with transcriptional amplification and invasion; and DNA repair mechanisms anchor the stress tolerance response. Pathways are annotated with representative Hallmarks of Cancer. Though not all hallmarks are displayed to avoid visual overcrowding. The Cell Survival module functionally subdivides into intrinsic apoptosis regulators (*BCL2*, *BCL2A1*, *BCL2L1*), NF-κB/IAP survival signaling (*BCL3*, *BIRC5*), and DNA damage checkpoints (*TP53*). Key drivers such as *EGFR* and *TP53* are positioned as central hubs, reflecting their roles in mediating and sustaining Proliferative Signaling and Genome Instability, respectively. Interconnections between pathways (e.g., RTKs → RAS–MAPK, RTKs → PI3K–AKT, RAS–MAPK → cell cycle, PI3K–AKT → apoptosis evasion, cytokine/inflammation → transcriptional amplification and invasion) are shown as canonical specific pathway-oriented knowledge edges. The biological rationale for each edge is detailed in Table S1.

To define the baseline architecture of the study, we first mapped the 102 selected consensus cancer genes onto a canonical oncogenic core network (Figure 1). This network illustrates how the selected genes organize into interconnected functional modules that collectively sustain malignant phenotypes. RTKs & Adaptors (e.g., *EGFR*, *ERBB2*, *MET*) act as upstream hubs, transmitting extracellular growth signals into the RAS–MAPK core and PI3K–AKT axis. The RAS–MAPK module—constitutively locked in an active state by the gain-of-function *KRAS* G12S mutation—links directly to Cell Cycle & Replication (e.g., *CCND1*, *CDK4*) and Transcriptional Amplifiers include canonical oncogenes (e.g., *MYC*) and contextual promoters (e.g., *STAT3*, *JUN*), collectively enforcing the Hallmarks of Sustaining Proliferative Signaling and Enabling Replicative Immortality.

In parallel, the PI3K–AKT axis functions as a critical survival node. In the specific context of A549 cells, this axis connects to Cell Survival to override the apoptotic potential of wild-type *TP53* while engaging anti-apoptotic effectors like *BCL3*. This active suppression ensures Resisting Cell Death and Deregulating Cellular Energetics, maintaining viability despite oncogenic stress. Cytokine/Inflammatory Signaling (e.g., *IL6*, *TGFB1*—contextual tumor promoters) feeds directly into transcriptional amplification and Invasion & ECM Remodeling (e.g., *CD44*, *MMP9*), linking Tumor-Promoting Inflammation to Activating Invasion & Metastasis, a connection that is particularly amplified by the *STK11* deficiency inherent to this cell line.

Finally, DNA Repair & Stress Tolerance (e.g., *ERCC2*, *MSH6*) anchors the hallmark of Genome Instability. Critically, this module is shaped by the loss-of-function *KEAP1* G333C mutation found in A549 cells. By disabling the “brake” on oxidative stress responses, this mutation creates a constitutive antioxidant shield that protects the tumor against therapeutic insults, maintaining a permissive state for oncogenic evolution.

### 2.2. Classification of Genes by Pathway and Global Centroid Dynamics

**Figure 2:**
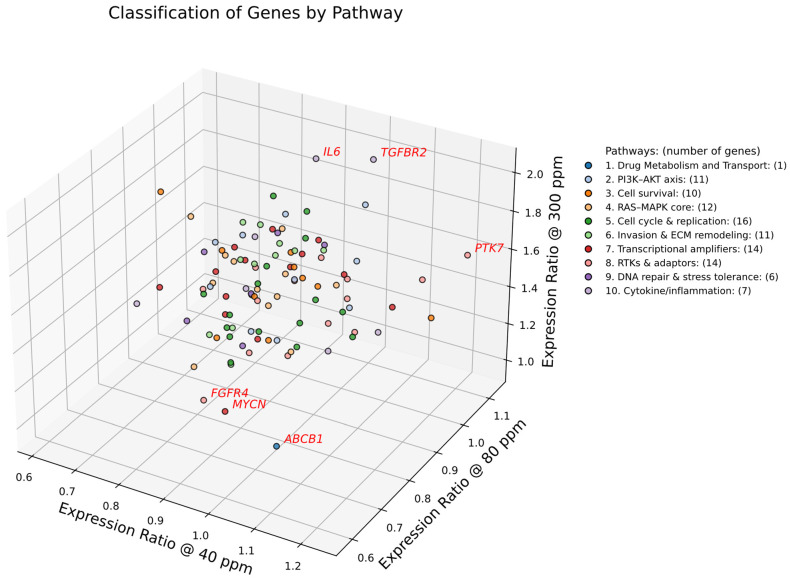
Three-dimensional classification of consensus cancer genes in A549 cells under variable deuterium concentrations. Scatter plot of 102 genes, each positioned by its expression ratios at 40 ppm (x-axis), 80 ppm (y-axis), and 300 ppm (z-axis), normalized to copy numbers at the 150 ppm control. Genes are color-coded by functional pathway, with the number of genes indicated in parentheses: drug metabolism and transport (*n* = 1), PI3K–AKT axis (*n* = 11), cell survival (*n* = 11), RAS–MAPK signaling (*n* = 11), cell cycle and replication (*n* = 16), invasion and extracellular matrix remodeling (*n* = 12), transcriptional amplifiers (*n* = 14), receptor tyrosine kinases and adaptors (*n* = 14), DNA repair and stress response (*n* = 5), cytokine/inflammatory signaling (*n* = 7). Genes highlighted in red (e.g., *TGFBR2*, *IL6*) are positioned farthest from the global centroid, marking the strongest expression responses to altered deuterium concentration. Pathway centroids are provided in Table 1. This visualization illustrates both the segregation of canonical oncogenic versus stress-response pathways and the identification of sentinel genes with heightened sensitivity to deuterium concentration change.

To map the gene expression landscape of A549 cells under deuterium stress, we plotted the 102 consensus genes in a three-dimensional expression space defined by their ratios at 40, 80, and 300 ppm relative to the 150 ppm control (Figure 2). Genes were color-coded by pathway, and their collective behavior was quantified by calculating pathway-specific centroids (Table 1). To identify outliers, we ranked all genes by their Euclidean distance from the Global Center. The top six genes: *TGFBR2*, *IL6*, *FGFR4*, *ABCB1*, *MYCN*, and *PTK7* were selected based on their pronounced divergence and a clear inflection point in the distance distribution (highlighted in red in Figure 2).

The quantitative analysis of pathway centroids (Table 1) reveals a monotonic increase in pathway-level gene expression correlating with deuterium concentration. The Global Center coordinates (0.884 → 0.940 → 1.436) serve as the system-wide baseline:

At 40 ppm, the global centroid (0.884) is well below unity, indicating that extreme deuterium depletion exerts a broad suppressive effect on the oncogenic expression.

At 80 ppm, expression remains dampened (0.940), reinforcing the concept of depletion as a “metabolic brake.”

At 300 ppm, the global centroid surges to 1.436, reflecting a robust, system-wide activation (~44% increase).

Using this global 1.436 value as a benchmark reveals distinct pathway sensitivities. Pathways exceeding this threshold at 300 ppm—including Cytokine/Inflammation (1.567), Cell Survival (1.476), and Invasion & ECM Remodeling (1.469)—are hyper-responsive to deuterium enrichment. In contrast, RTKs & Adaptors (1.377) and Cell Cycle & Replication (1.371) respond more modestly, falling below the global average. Notably, Drug Metabolism and Transport (*ABCB1*) exhibit a unique trajectory with extreme suppression at 80 ppm (0.585), diverging sharply from the global trend. These results indicate that A549 cells adapt to deuterium stress by suppressing energetically costly programs (like drug efflux) under depletion, while enrichment drives a coordinated reprogramming that favors survival, invasion, and inflammation.

### 2.3. Density-Based Spatial Clustering

**Figure 3:**
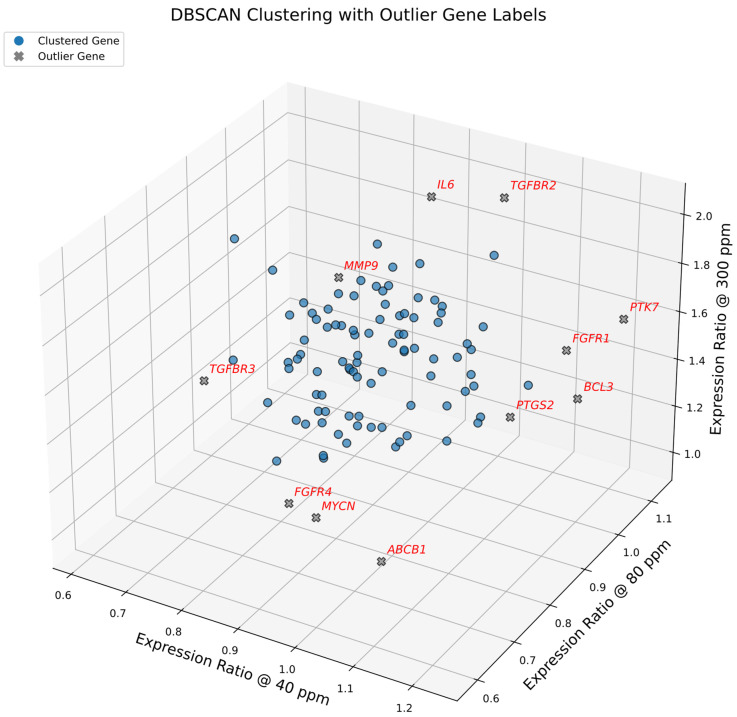
DBSCAN of deuterium-responsive gene expression in A549 cells. Three-dimensional scatter plot showing expression ratios of 102 consensus cancer genes at 40, 80, and 300 ppm deuterium, normalized to the 150 ppm control. Each axis represents the expression ratio at one concentration. DBSCAN analysis (ε = 0.13, min_samples = 5) separates the dense gene expression core (blue circles) from 11 sentinel outlier genes (gray x symbols, labeled in red italics). Outliers exhibit trajectories that diverge strongly from pathway averages, highlighting stress-sensitive regulators and context-dependent modulators. Notably, these outliers map to distinct Hallmarks of Cancer: *ABCB1* (drug resistance; Resisting Cell Death), *FGFR4* (growth factor signaling; Sustaining Proliferative Signaling), *MYCN* (transcriptional amplification; Enabling Replicative Immortality), *IL6* and *TGFBR2* (inflammation and invasion; Tumor-Promoting Inflammation and Activating Invasion & Metastasis). Cells were cultured for 72 h under the indicated conditions.

To complement centroid-based identification of expression-sensitive genes, we applied DBSCAN to the 3D expression space defined by relative expression ratios at 40 ppm, 80 ppm, and 300 ppm deuterium, as shown in Figure 3. A 5-distance plot (Figure S1) was used to select the ε parameter, consistent with the standard practice of setting *min_samples* = 5. This choice balances sensitivity to small gene expression modules with robustness against noise, enabling detection of biologically meaningful outliers in the 102-gene dataset. Unlike centroid-based methods that emphasize spherical pathway-level coherence, DBSCAN identifies density-based outliers with unique expression ratios, independent of canonical pathway clustering. These outliers may represent representative responders or context-specific regulators whose behavior diverges from pathway averages, offering additional insight into deuterium-dependent gene expression heterogeneity.

To visually summarize the directional behavior of gene expression ratios across three deuterium concentrations (40, 80, 300 ppm), we assigned each outlier a pattern symbol triplet (e.g., → ↓ ↗) based on its relative expression values. These symbols reflect the magnitude and direction of change using a biologically interpretable scale: ↑ for strong upregulation (>1.37), ↗ for moderate upregulation (1.17–1.37), **→** for stable expression (0.82–1.17), **↙** for moderate downregulation (0.70–0.82), and **↓** for strong downregulation (<0.70). The thresholds were rigorously selected based on statistical distributions and centroid benchmarks (e.g., the 1.37 upper threshold aligns with the lowest centroid of upregulated clusters at 300 ppm).

### 2.4. Pathway-Specific Analysis of DBSCAN Outlier Genes

To elucidate the biological relevance of the 11 outlier genes identified by DBSCAN (ε = 0.13), we grouped them by functional pathway and analyzed their expression dynamics.

### 2.5. Drug Metabolism and Transport

*ABCB1* (→ ↓ ↗) encodes P-glycoprotein, a member of the ATP-Binding Cassette (ABC) transporter superfamily. It functions as an ATP-powered efflux pump that actively exports a wide range of hydrophobic molecules, including chemotherapeutic agents, making it a central mediator of multidrug resistance (MDR). In our dataset, *ABCB1* exhibited a non-linear response: mild induction at 40 ppm (+12%), suppression at 80 ppm (−42%) (with a small 7% error margin), and reactivation at 300 ppm (+27%). This distinct suppression at 80 ppm is particularly significant and identifies *ABCB1* as a sentinel gene highly sensitive to moderate deuterium depletion.

### 2.6. Cell Survival

*BCL3* (↗ → ↑) is an IκB-like protein that modulates NF-κB signaling, promoting transcription of anti-apoptotic genes and enabling tumor survival under stress. It is frequently upregulated in hematologic and solid malignancies. In this dataset, *BCL3* exhibited progressive upregulation across all deuterium concentrations, consistent with a robust survival response. Its low total error reinforces the reliability of this measurement and supports its role as a transcriptional amplifier of cell survival under deuterium concentration change.

### 2.7. RTKs and Adaptors

This functional group highlights a striking divergence in receptor sensitivity. *FGFR1* (→ → ↑) exhibited stable expression at physiological levels but surged at 300 ppm (+57%), indicating a threshold-dependent response to deuterium enrichment. In sharp contrast, *FGFR4* (↙ ↙ →) was consistently suppressed at 40 and 80 ppm (~−19%), suggesting that deuterium depletion dampens specific growth factor receptivity. *PTK7* (↗ → ↑), an orphan receptor involved in Wnt/Planar Cell Polarity signaling, displayed a progressive upward trend across all concentrations. Together, these profiles demonstrate that while enrichment fuels broad receptor activation (*FGFR1*, *PTK7*), depletion selectively compromises specific signaling nodes (*FGFR4*).

### 2.8. Cytokine and Inflammation

*IL6* (→ → ↑), *PTGS2* (↗ → ↑), *TGFBR2* (→ → ↑), and *TGFBR3* (↓ → ↗) are key regulators of inflammation and immune modulation. *IL6* and *PTGS2* promote tumor growth, angiogenesis, and immune suppression, with *IL6* showing strong induction at 300 ppm (+99%). *TGFBR2* and *TGFBR3* mediate TGF-β signaling, which plays context-dependent roles in cancer progression. *TGFBR2* was strongly induced at 300 ppm (+105%), while *TGFBR3* showed distinct suppression at 40 ppm (−36%) followed by recovery. These patterns suggest that deuterium enrichment amplifies inflammatory signaling, contributing to Tumor-Promoting Inflammation.

### 2.9. Invasion and ECM Remodeling

*MMP9* (↓ → ↑) encodes a matrix metalloproteinase that degrades extracellular matrix components, facilitating tumor invasion. It was suppressed at 40 ppm (−31%) and progressively induced at higher concentrations. As a driver of Activating Invasion & Metastasis, its suppression under depletion reflects a dose-dependent inhibition of invasive behavior.

### 2.10. Transcriptional Amplifiers

*MYCN* (→ ↙ →) is a member of the *MYC* family, regulating cell cycle and metabolism. It showed mild suppression at 80 ppm (−24%) followed by stability at 300 ppm (+1%). As a master regulator, *MYCN*’s modulation under deuterium stress may have cascading effects on downstream oncogenic programs.

### 2.11. Core Pathway Stability

In the current dataset of 102 genes, four pathways, like Cell cycle & replication, DNA repair & stress tolerance, PI3K–AKT axis, and RAS–MAPK core, contained no outlier genes. This consistency shows that all genes in these core oncogenic programs responded in a moderate, coordinated manner to deuterium concentration changes, indicating that these pathways act as stable, coherent modules. In contrast, heterogeneous pathways like Cytokine and Inflammation showed larger variations, producing multiple outliers.

### 2.12. Symbolic Clustering of Gene Expression Patterns

**Figure 4:**
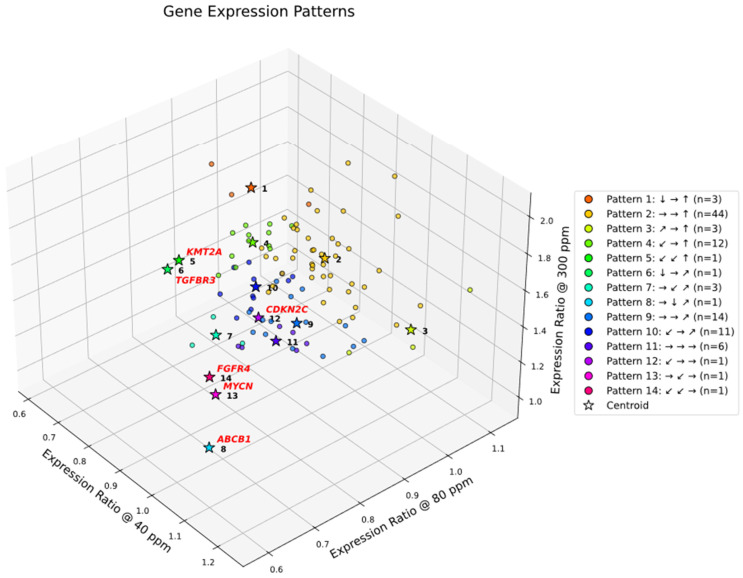
Symbolic clustering of gene expression trajectories under graded deuterium concentrations. Three-dimensional scatter plot showing expression ratios of 102 consensus cancer genes at 40, 80, and 300 ppm deuterium, normalized to the 150 ppm control. Genes are color-coded by their symbolic triplet pattern (e.g., ↓ → ↑), with cluster centroids indicated by color-coded stars. Patterns are ordered by their expression ratio at 300 ppm (highest to lowest). The legend specifies each triplet and the number of genes (n) per group. Only 14 of 125 theoretical triplets are represented, with Pattern 2 (→ → ↑; *n* = 44) emerging as the dominant trajectory. See Results for detailed interpretation. Genes highlighted in red (e.g., *ABCB1*, *FGFR4*, *MYCN*) represent unique singleton trajectories, diverging from canonical clusters and marking representative regulators of drug resistance, growth factor signaling, and gene expression amplification.

In this section, we analyze gene expression patterns in the 102-gene dataset using the discretized thresholds introduced above. Symbolic clustering across deuterium concentrations reveals a striking sparsity in realized gene expression patterns. Although the symbolic encoding scheme allows for 125 theoretical combinations, only 14 distinct patterns were observed (cf. Figure 4). This >90% reduction in theoretical complexity reflects strong biological constraints, suggesting that A549 cells converge onto a limited set of coordinated regulatory programs to manage deuterium stress.

### 2.13. Dominant Gene Expression Patterns

The distribution of genes across these 14 patterns is highly skewed. Most genes converge into just three dominant clusters, with Pattern 2 emerging as the overarching gene expression program.

Pattern 2 (→ → ↑): This dominant group (*n* = 44) represents genes that remain stable under depletion but surge under enrichment. It encapsulates the “Enrichment as Accelerator” phenotype, comprising nearly half the dataset and driving the Hallmarks of Resisting Cell Death, Sustaining Proliferative Signaling, and Tumor-Promoting Inflammation.

Minor Clusters: Two other patterns (Patterns 4 and 9) contain 12 and 14 genes, representing alternative regulatory modes. Patterns 1, 3, 7, and 11 contain between 3 and 6 genes.

### 2.14. Singleton Outliers and Methodological Consensus

In contrast to the dominant groups, six genes: *ABCB1*, *CDKN2C*, *FGFR4*, *KMT2A*, *MYCN*, and *TGFBR3*, exhibit unique symbolic patterns not shared by any other gene. Importantly, four of these six singleton genes align with the outliers identified by DBSCAN, reinforcing their status as biologically distinct gene expression entities (Figure 4). For instance, *ABCB1* and *FGFR4* consistently diverge from the population mean, spanning critical hallmarks of Multidrug Resistance and Growth Factor Signaling.

Comparative Outlier Detection and Dataset Refinement

**Figure 5:**
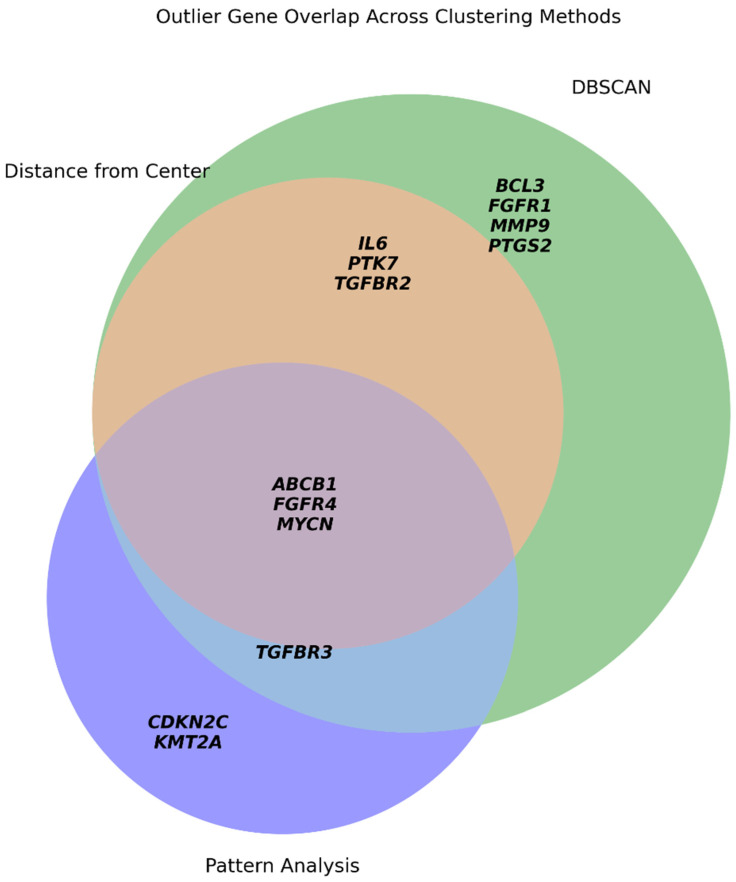
Overlap of gene expression outlier genes identified by three clustering strategies. Venn diagram showing the intersection of genes classified as outliers by three independent approaches: (1, orange) Euclidean distance from the global centroid; (2, green) DBSCAN; and (3, violet) unique singleton symbolic patterns. The overlap defines a consensus core of highly divergent genes (e.g., *ABCB1*, *MYCN*, *FGFR4*), while also illustrating that DBSCAN captures the broadest set of sentinel responders.

We evaluated gene expression outliers using three complementary approaches: global centroid distance, DBSCAN, and symbolic pattern analysis (Figure 5).

Centroid-based: Flagged six genes, all of which were re-confirmed by DBSCAN.

Symbolic Analysis: Overlapped with four DBSCAN-defined genes and identified unique trajectories for *CDKN2C* and *KMT2A*.

DBSCAN: Proved the most sensitive method, identifying the “core” outliers captured by other methods, plus five additional context-specific regulators.

This comparison demonstrates that while distance-based metrics provide baseline selectivity, DBSCAN offers superior sensitivity in detecting gene expression divergence. Accordingly, for the subsequent GMM clustering, we excluded the full set of DBSCAN-defined outliers to improve cluster resolution and biological interpretability, reducing the dataset to 91 genes.

In summary, the symbolic clustering framework delineates a biologically constrained gene expression landscape, characterized by a limited set of dominant high-expression patterns and punctuated by distinct outliers. The sparsity of realized patterns, the centrality of Pattern 2, and the functional relevance of singleton genes highlight the importance of integrated clustering and pathway-level interpretation in future analyses. Nonetheless, propagated measurement error in the current dataset may reach 10–15%, potentially causing genes near classification boundaries to overlap with adjacent patterns. This ambiguity (see Figure S2) highlights the need for complementary unsupervised clustering approaches. Moreover, the five-category classification scheme may be overly rigid for capturing extreme expression levels; for example, it assigns the same symbol (*↑*) to 40% and 100% overexpression. To complement the symbolic classification, we next applied unsupervised clustering using Gaussian Mixture Models (GMM), which provide a probabilistic framework for identifying continuous gene expression variation and resolving ambiguous boundary assignments. Gaussian Mixture Models are particularly suited to this dataset because they treat expression ratios as overlapping probability distributions, allowing genes with propagated error near symbolic boundaries to be assigned probabilistically rather than rigidly. This flexibility captures subtle shifts and resolves the ambiguity inherent in discrete symbolic categories.

### 2.15. GMM Clustering 91 Genes with Tied Covariance Ellipsoids

We evaluated Gaussian Mixture Models (GMMs) using silhouette analysis across seven candidate cluster counts (*k* = 2 to 9), where *k* denotes the number of components the algorithm attempts to identify (cf. Figure S3). Each cluster ideally captures a distinct gene expression pattern under varying deuterium concentrations.

### 2.16. Statistical Model Selection

A critical observation from the silhouette analysis is that all scores remained below 0.5. In clustering topology, a coefficient < 0.5 indicates that the data structure is not characterized by distinct, widely separated “islands,” but rather represents a continuous biological gradient with overlapping boundaries. Consequently, the silhouette score alone was insufficient to determine the optimal partition, requiring a balance between statistical separation and biological resolution.

Rejection of *k* = 2: Although the tied covariance model with *k* = 2 yielded the highest numerical silhouette score, inspection revealed this solution was uninformative, providing only a trivial binary partition (high vs. low expression at 300 ppm) that masked pathway-specific dynamics.

Selection of *k* = 4 and *k* = 6: The tied covariance model with *k* = 4 yielded the second-highest score, closely followed by *k* = 6. This configuration achieved a favorable Bayesian Information Criterion (BIC = −438) and log-likelihood (LogLik = 267), suggesting an optimal balance between model fit and complexity.

### 2.17. Model Robustness and Sensitivity Analysis

While full covariance models produced higher LogLik values, their substantially worse BIC scores pointed to overfitting, justifying the use of tied covariance. Overall, GMM clustering (silhouette = 0.320) outperformed *k*-means (silhouette = 0.255), confirming that the probabilistic, ellipsoid-based boundaries of GMM better capture the complex covariance structure of oncogenic gene expression than the spherical assumptions of *k*-means.

To assess clustering robustness, we performed a leave-one-out sensitivity analysis comparing GMMs with 4 and 6 clusters (GMM-4 and GMM-6, cf. Table S2). For each gene, we computed the Adjusted Rand Index (ARI) after its removal. The average ARI difference was −0.043, indicating that GMM-6 is marginally more stable overall. However, gene-specific impacts varied:

Stabilizers: Genes such as *BIRC5* and *MUC1* stabilized the GMM-4 solution, reflecting their roles in core cell cycle/survival modules, cf. Table S2.

Destabilizers: Genes like *AKT2* and *BCL2A1* destabilized GMM-4, suggesting they sit at the boundaries of broader clusters and require the higher resolution of GMM-6 to be accurately partitioned, cf. Table S2.

Instability of *k* = 9: Notably, the 9-cluster solution proved unstable; removal of a single gene triggered complete cluster rearrangement, confirming that *k* = 9 over-fragmented the data into artificial groups.

### 2.18. Cluster Visualization and Boundary Drivers

**Figure 6:**
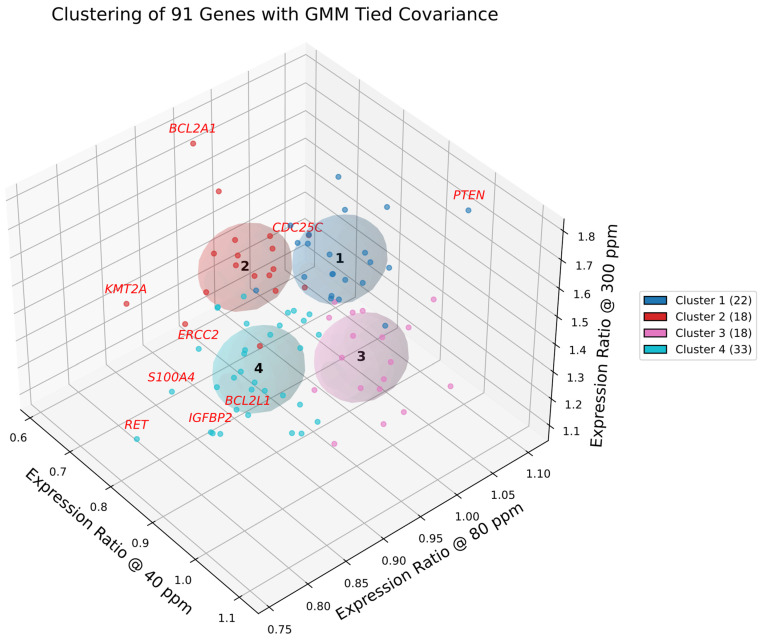
Gaussian mixture clustering of deuterium-responsive genes in A549 cells. Three-dimensional scatter plot of 91 genes, each positioned by its expression ratios at 40, 80, and 300 ppm relative to the 150 ppm control. Gaussian mixture modeling with tied covariance ellipsoids (*k* = 4) partitions the dataset into four clusters, shown with color-coded membership and corresponding covariance ellipsoids. The legend reports the number of genes per cluster. Genes with the greatest Mahalanobis distance from their cluster centroids (e.g., *BCL2A1*, *PTEN*) are highlighted in red, denoting boundary outliers with divergent gene expressions.

**Figure 7:**
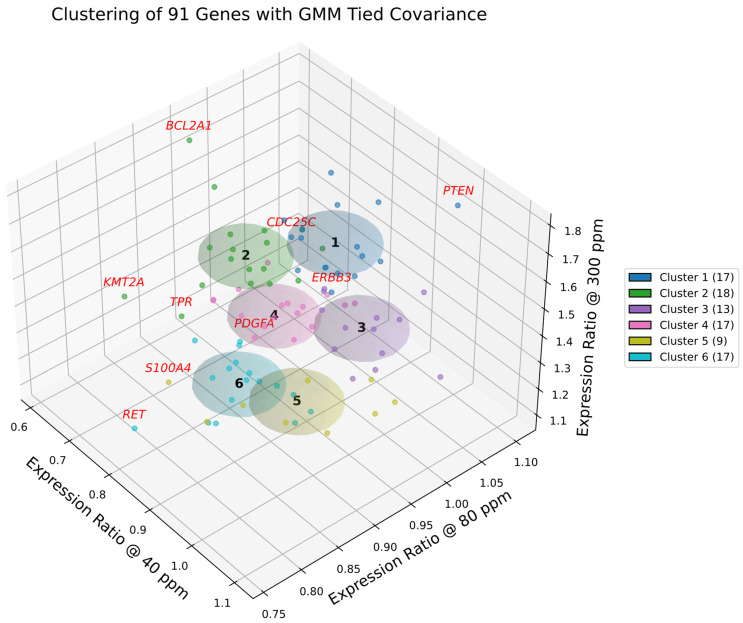
Gaussian mixture clustering of deuterium-responsive genes in A549 cells. Three-dimensional scatter plot of 91 genes positioned by their relative expression ratios at 40, 80, and 300 ppm compared to the 150 ppm control. Gaussian mixture modeling with tied covariance ellipsoids (*k* = 6) partitions the dataset into six optimal clusters, selected based on silhouette analysis and biological coherence. Genes and covariance ellipsoids are color-coded by cluster membership, with cluster sizes reported in the legend. Genes exceeding a Mahalanobis distance of 2.42 from their cluster centroid (e.g., *BCL2A1*, *PTEN*) are highlighted in red, denoting boundary outliers that extend beyond the canonical definition of the gene expression module.

Figure 6 and Figure 7 present the results of GMM-4 and GMM-6 applied to the curated set of 91 cancer-related genes. In the selected model, each cluster is represented by a shared covariance ellipsoid centered at its coordinate mean. To assess the confidence of assignments, we computed the Mahalanobis distance of each gene from its cluster centroid, cf. Table S2. Unlike Euclidean distance, Mahalanobis distance accounts for the covariance structure, penalizing directions of low variance. Genes with high Mahalanobis distances (e.g., *PTEN*, *CDC25C*, *ERBB3*, *BCL2A1*) were identified as boundary drivers—genes that are statistically peripheral to their cluster yet biologically critical for defining the cluster’s functional edge (e.g., balancing tumor suppression with amplification).

### 2.19. Cluster Transitions and Heterogeneity (GMM-4 to GMM-6)

**Figure 8:**
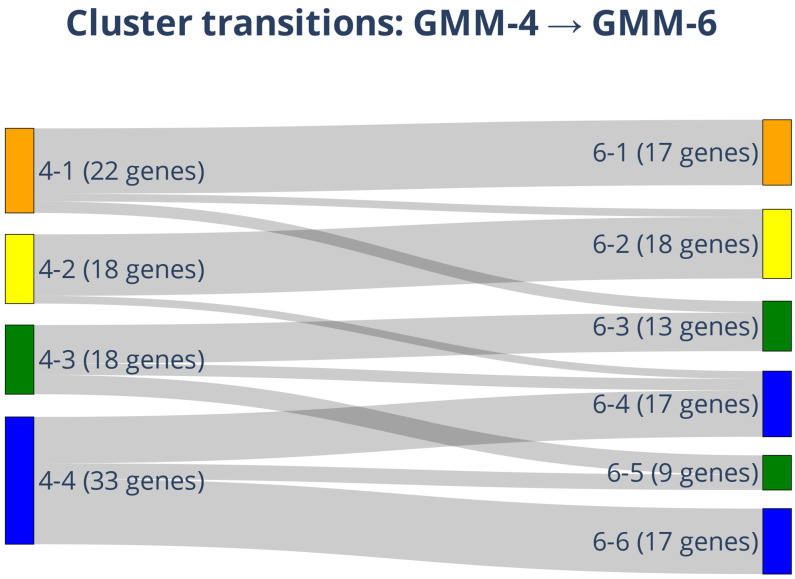
Sankey diagram of gene cluster transitions between GMM-4 and GMM-6 models. Sankey diagram illustrating how gene memberships redistribute when Gaussian mixture modeling is expanded from four clusters (left) to six clusters (right). Each node represents a cluster, labeled by ID (e.g., 4-1) and gene count. Colors correspond to the original GMM-4 cluster assignments, and flow widths are proportional to the number of genes transitioning between clusters. Increasing model granularity partitions the large, heterogeneous GMM-4 Cluster 4 (blue) into three distinct functional submodules (GMM-6 Clusters 4, 5, and 6), thereby separating invasive and proliferative drivers from basal maintenance genes.

To visualize how increasing model granularity redistributes genes, we mapped the transitions from GMM-4 to GMM-6 using a Sankey diagram (cf. Figure 8). The analysis revealed clear patterns of stability and fragmentation:

Stable Modules: Genes assigned to GMM-4 clusters 4-1 and 4-2 (22 and 18 genes, respectively) largely remained together, mapping almost exclusively into GMM-6 clusters 6-1 and 6-2. This indicates that these gene expression programs (associated with Cellular Regulation and KRAS Effector signaling) are robust across model resolutions.

Fragmented Heterogeneity: In contrast, the largest GMM-4 cluster (4-4, *n* = 33) was partitioned into multiple GMM-6 clusters (6-4, 6-5, and 6-6). This dissociation unmasked critical heterogeneity: it separated the Invasive/Plasticity drivers (*TGFB1*, Cluster 5) and Growth Factors (*PDGFA*, Cluster 4) from the Basal Stability genes (*BIRC5*, Cluster 6). The proportional widths of the flows emphasize that while some gene expressions are robust, others fragment into finer substructures, proving that the six-cluster resolution is necessary to distinguish Invasion programs from general survival mechanisms.

### 2.20. Composition and Biological Identity of GMM-6 Clusters

Gaussian mixture modeling with tied covariance ellipsoids (*k* = 6), validated by silhouette analysis, delineated six distinct gene groups whose centroid trajectories across deuterium concentrations define the gene expression landscape of A549 cells.

Clusters 1 and 2 showed the strongest induction at 300 ppm (cf. Figure 7), representing stress-responsive proliferative and survival modules:

Cluster 1 integrated cell cycle regulators and DNA repair factors, with boundary outliers (*PTEN*, *CDC25C*, *ERBB3*) marking the tension between tumor suppression and receptor-driven proliferation. Canonical oncogenes (*MYC*, *STAT3*, *TERT*) reinforced its proliferative bias within the context of a wild-type *TP53* axis.

Cluster 2 encompassed transcriptional amplifiers and apoptosis regulators, with outliers (*BCL2A1*, *KMT2A*, *TPR*) highlighting survival stability. Importantly, this cluster contained the core RAS effector *RAF1* alongside *EGFR*, underscoring its role as the primary signaling engine driven by the G12S mutation.

Cluster 3 was cohesive, balancing oncogenic drivers (*ERBB2*, *JUN*, *IGF1*) with the tumor suppressor *TP53*. This module reflects a signaling core where proliferative drive is counterweighted by suppressive control. While basal transcriptional load is driven by oncogenic RAS, the co-clustering of *TP53* with homeostatic regulators confirms that A549 cells retain a competent, stress-responsive p53 axis. This module preserves canonical regulatory architecture, enabling a coordinated, though ultimately overwhelmed, response to deuterium modulation.

Cluster 4 contained accessory growth factor and stress response genes, with *PDGFA* defining its proliferative edge. Oncogenes (*NRAS*, *MET*, *LYN*) were present alongside apoptosis regulators (*BCL2*, *FAS*), highlighting a dual role in growth signaling and stress adaptation distinct from the primary RAS driver in Cluster 2.

Cluster 5 was enriched for ECM remodeling and invasion, with *S100A4* marking the invasive boundary. Key members included the oncogene *HRAS* alongside the tumor promoter *TGFB1*. The enrichment of these plasticity drivers is consistent with the phenotype of *STK11* (LKB1) deficiency inherent to A549 cells, identifying this cluster as the primary engine of microenvironmental adaptation and metastatic potential.

Cluster 6 grouped cell cycle and repair genes that remained relatively stable across conditions, with *RET* delineating its invasion-linked outlier. Oncogenes (*AKT2*, *CDK4*, *FGFR3*) were central, together with survival factors such as *BIRC5* (Survivin). This defines a “Basal Maintenance” cluster that sustains viability even when more plastic modules (Cluster 5) are suppressed.

Together, these clusters reveal a structured landscape in which proliferative and survival modules dominate the stress response, while invasion and repair pathways provide adaptive support. Outlier genes stretch cluster boundaries, identifying the most stress-responsive drivers of instability. Notably, several highlighted genes—including *TP53*, *EGFR*, and *RET*—are clinically relevant in the A549 LUAD cell line, underscoring the translational importance of these clustering results.

## 3. Discussion

### 3.1. Deuterium as a Dual Regulator of Oncogenic Physiology

Recent work has highlighted that intracellular deuterium concentration is not a passive variable but an active regulator of cellular physiology [14], with both depletion and enrichment exerting measurable effects on cancer biology. Reviews have shown that deuterium enrichment can drive oxidative stress, metabolic rewiring, and increased gene expression [14], while deuterium depletion has been reported to suppress tumor growth in vitro [9], in vivo [6], and in clinical settings [9,15]. Our findings that A549 cells exhibit global downregulation under depletion (40–80 ppm) but a marked increase in gene expression under enrichment (300 ppm) are consistent with these observations. We extend this knowledge by providing pathway-level resolution of the adaptive programs engaged by cancer cells. In particular, the preferential upregulation of Tumor-Promoting Inflammation, PI3K–AKT survival, and invasive remodeling pathways under enrichment suggests that deuterium availability couples directly to redox balance, metabolic flux, and stress-response gene expression networks, thereby shaping tumor cell fitness.

### 3.2. Clinical Resonance and the A549 Representative Model

Our cell-level observations resonate with clinical and translational studies of deuterium-depleted water (DDW), which consistently demonstrate antiproliferative effects. However, these results must be contextualized within the specific genetic architecture of the A549 model. As a *KRAS/STK11/KEAP1* co-mutant line with functional *TP53*, A549 cells represent an aggressive, “immune-cold” phenotype often resistant to standard therapies.

The suppression of *ABCB1* (P-glycoprotein) and *MMP9* observed under depletion provides mechanistic evidence that deuterium concentration change can bypass these hardwired defenses. This implies that the clinical benefits of DDW reported in systematic reviews may arise not only from general stress but also from selective dismantling of resistance networks—such as NRF2-driven antioxidant shields—that aggressive, metabolically rewired tumors rely on for survival.

### 3.3. Concentration-Dependent ABCB1 Expression Defines an Active Concentration Window

The suppression of *ABCB1* reveals a highly specific, concentration-dependent vulnerability in the A549 antioxidant defense system. Critically, this response is not linear: while *ABCB1* is suppressed by 42% at 80 ppm, it is slightly induced (+12%) at 40 ppm. This non-linearity defines a distinct active therapeutic window for deuterium depletion.

In the context of the A549 *KEAP1* G333C loss-of-function mutation, which drives constitutive NRF2 activity, this “V-shaped” response suggests distinct adaptive thresholds. We hypothesize that moderate depletion (80 ppm) functions as a metabolic brake sufficient to destabilize the energetic support for NRF2-driven transcription, effectively bypassing the genetic defect. By contrast, extreme depletion (40 ppm) appears to exceed a stress threshold, potentially triggering a compensatory survival alarm that re-engages drug resistance mechanisms despite the metabolic constraint. This finding challenges the “lower is better” paradigm, suggesting that maximizing therapeutic efficacy against multidrug resistance requires targeting the specific deuterium concentration window (e.g., 80 ppm) where the metabolic cost of resistance exceeds the cell’s adaptive capacity without triggering a rebound response.

### 3.4. Resolving Latent Heterogeneity: The Biological Necessity of GMM-6

While the four-component model (GMM-4) captured broad biological programs such as proliferation, the transition to the six-component model (GMM-6) was essential to resolve latent phenotypic heterogeneity. The statistical stability (ARI) of both models masks a critical biological trade-off: GMM-4 obscures the distinction between basal maintenance and active plasticity.

Sankey transition analysis demonstrates that this refinement is not random. The fragmentation of the large GMM-4 Cluster 4 into GMM-6 Clusters 4, 5, and 6 unmasked distinct adaptive strategies:

Cluster 4 retained growth factor-driven proliferation (*PDGFA*, *NRAS*).

Cluster 5 emerged as a distinct module driving phenotypic plasticity (*TGFB1*, *S100A4*).

Cluster 6 isolated stable, basal survival machinery (*AKT2*, *BIRC5*).

This separation is biologically crucial, particularly given the *STK11*-deficient status of A549 cells, which drives invasion. It reveals that the invasive phenotype (Cluster 5) is distinct from basal survival (Cluster 6) and, importantly, is far more sensitive to deuterium depletion. By resolving these modules, we demonstrate that deuterium stress does not simply suppress the cell uniformly; rather, it selectively disengages the high-energy programs required for *Activating Invasion and Metastasis* while leaving the core survival machinery relatively intact.

### 3.5. Biological Coherence of GMM-Derived Clusters and Pathway Enrichment

In contrast to widely used gene expression stratification methods such as consensus clustering, non-negative matrix factorization (NMF), or single-cell trajectory inference, our symbolic clustering approach is not intended to define stable molecular subtypes or extract latent programs. Instead, symbolic clustering provides a coarse-grained, categorical representation of within-gene trajectories across variable deuterium concentrations. Each gene’s expression profile is compressed into a triplet of intuitive symbols (e.g., ↓ → ↑), serving as a visual shorthand for directional behavior.

This symbolic encoding complements the probabilistic assignments generated by the Gaussian mixture model (GMM) clustering by highlighting how expression changes unfold across conditions, rather than only which cluster a gene belongs to. The result is a compact, biologically interpretable map that facilitates rapid recognition of coordinated regulatory programs, identification of sentinel outliers, and cross-pathway comparability. Thus, symbolic clustering functions as a visualization and interpretive overlay, offering accessibility and interpretability beyond conventional clustering frameworks.

The six GMM clusters exhibit distinct gene expression profiles across exposure levels, with centroid coordinates stratified by decreasing expression at 300 ppm. This stratification reflects biologically meaningful modules, as confirmed by Kyoto Encyclopedia of Genes and Genomes (KEGG) and Reactome pathway enrichment.

The pathway categories shown in Figure 1 and Figure 2 represent canonical bins used for visualization, where genes are grouped into broad functional classes such as PI3K–AKT axis, RAS–MAPK signaling, cell cycle and replication, or invasion/ECM remodeling. In contrast, the pathway labels assigned to clusters below derive from enrichment analysis of the specific gene sets within each cluster. This approach sometimes introduces more granular or mechanistic terms (e.g., JAK–STAT signaling, FoxO signaling, EMT, hypoxia, focal adhesion) that refine but do not replace the canonical categories. For example, *STAT3* in Cluster 1 supports annotation as JAK–STAT signaling, even though in Figure 2 it was grouped under cytokine/inflammatory signaling. Similarly, *TGFB1* and *S100A4* in Cluster 5 justify annotation as Epithelial–Mesenchymal Transition (EMT), which is a mechanistic subdivision of invasion/ECM remodeling. Thus, the cluster nomenclature should be interpreted as functional refinements of the broader bins in Figure 2, providing higher-resolution modules that capture context-specific behavior under deuterium concentration change.

### 3.6. Pathway and Hallmark Coherence Among High-Confidence Cluster Members

**Cluster 1: Cellular Regulation and Genomic Stability**

17 genes, see Table 2.

*Pathways:* Cell Cycle, PI3K–AKT axis, DNA Repair, JAK–STAT signaling

Hallmarks: Genome Instability & Mutation, Enabling Replicative Immortality

This cluster exhibits the most dramatic response to deuterium enrichment, rising to an expression ratio of 1.66 at 300 ppm. It integrates the *TP53*-responsive phosphatase *CDC25C* and the tumor suppressor *PTEN* with potent transcriptional amplifiers from the Core Network (Figure 1), specifically *MYC* and *STAT3*. In A549 cells, which retain a wild-type *TP53* axis, this cluster represents a functional checkpoint response coupled with a proliferative surge. The co-clustering of *ERBB3* (RTK adaptor) with *MYC* implies that deuterium enrichment simultaneously fuels upstream growth signals and downstream replication programs, engaging the p53 machinery (*PTEN*, *CDC25C*) to maintain genomic integrity under proliferative stress.

**Cluster 2: KRAS Effector and Hypoxic Response Module**

18 genes, see Table 2.

*Pathways:* RAS–MAPK signaling, Hypoxia, Focal Adhesion

Hallmarks: Sustaining Proliferative Signaling, Activating Invasion & Metastasis

Cluster 2 reveals a critical metabolic vulnerability in the *KRAS*-driven engine. It displays the steepest suppression at 40 ppm (0.792). Biologically, this cluster contains *RAF1* (c-Raf), the direct downstream effector of mutant *KRAS*, alongside the upstream receptor *EGFR*. While the G12S mutation constitutively activates *KRAS*, the monotonic response of this cluster indicates that the effector machinery (*RAF1*, *HSP90AB1*) is highly sensitive to deuterium deprivation. The inclusion of *HIF1A* and the invasion marker *CD44* suggests that depletion imposes a powerful metabolic brake on proliferation and hypoxic adaptation, with suppression magnitude directly correlating with depletion level.

**Cluster 3: TP53 Homeostatic Hub**

13 genes, see Table 2.

*Pathways:* p53 Signaling, Cell Cycle, FoxO Signaling

Hallmarks: Evading Growth Suppressors, Deregulating Cellular Energetics

Cluster 3 is characterized by stability at 40 and 80 ppm, acting as a homeostatic core. The central node is *TP53*. Unlike p53-mutant subtypes, where DNA damage responses are uncoupled from growth arrest, the clustering of *TP53* with growth drivers—including *CCND1*, *ERBB2*, and *IGF1*—confirms that A549 cells retain a competent, stress-responsive p53 axis. While basal transcriptional load is driven by oncogenic *KRAS*, this module preserves canonical regulatory architecture, balancing growth signaling (*IGF1*, *JUN*) with suppression until overwhelmed at 300 ppm. The observed stability suggests that moderate depletion is perceived not as a genotoxic crisis but as a metabolic shift, allowing basal p53 surveillance to persist.

**Cluster 4: Accessory Growth Signals (“Co-Conspirators”)**

17 genes, see Table 2.

*Pathways:* MAPK signaling, Apoptosis, Nucleotide Excision Repair

Hallmarks: Sustaining Proliferative Signaling, Resisting Cell Death

This cluster is suppressed under depletion (0.820 at 40 ppm), representing the accessory signaling arm. It contains *NRAS* and *MET*. In *KRAS*-mutant tumors, wild-type isoforms like *NRAS* often act as co-conspirators to support the phenotype. The sensitivity of this cluster highlights synthetic vulnerability: while mutant *KRAS* is constitutively active, supporting growth factor networks (*MET*) and anti-apoptotic reserves (*BCL2*) are energetically costly. Their downregulation under depletion suggests they can be decoupled from the primary driver when energetics are constrained. The presence of *XPC* (DNA repair) and *ETS1* (transcription factor) further links this cluster to maintenance of the oncogenic state outlined in Figure 1.

**Cluster 5: Invasion and Phenotypic Plasticity (STK11-Linked)**

9 genes, see Table 2.

*Pathways:* TGF-β signaling, EMT

Hallmarks: Unlocking Phenotypic Plasticity, Activating Invasion & Metastasis

This cluster is critical for the *STK11*-deficient phenotype of A549 cells, associated with aggressive invasion. Enriched for *TGFB1* (contextual tumor promoter) and the metastasis driver *S100A4*, this module drives EMT. Its trajectory is distinct, showing a dip at 80 ppm relative to 40 ppm, suggesting non-linear sensitivity where specific concentrations disrupt signaling thresholds for plasticity. The presence of *HRAS* and *BCL2L1* (Bcl-xL) indicates that this invasive program is tightly coupled to survival mechanisms distinct from basal machinery.

**Cluster 6: Basal Maintenance and Survival Reserve**

17 genes, see Table 2.

*Pathways:* PI3K–AKT signaling, Cell Cycle, DNA Repair

Hallmarks: Resisting Cell Death, Deregulating Cellular Energetics

Cluster 6 exhibits the lowest induction at 300 ppm (1.180), defining it as a basal maintenance reserve. It contains key Core Network hubs: *AKT2* and *CDK4*. In the context of *STK11* deficiency, the mTOR/AKT pathway is often hyperactivated. The relative stability of this cluster suggests that constitutive survival signaling is robust. However, suppression at 40 ppm (0.830) indicates that even basal reserves are not immune to extreme depletion. The presence of *BIRC5* (Survivin)—typically repressed by wild-type *TP53*—suggests that oncogenic signaling overrides the p53 brake to ensure viability, while *ERCC2* maintains background DNA repair.

Table 2 summarizes the key characteristics of each gene cluster identified by Gaussian Mixture Model (GMM-6) analysis of A549 lung adenocarcinoma cells under varying deuterium concentrations. Each row represents a distinct cluster, defined by its functional annotation, centroid coordinates (mean relative gene expression at 40, 80, and 300 ppm deuterium), and the list of genes assigned to that cluster.

**Table 2:** Summary of GMM-6 Cluster Centroids, Functional Annotations, and Gene Composition in A549 Cells Under Variable Deuterium Concentrations.

Cluster	Function	40 ppm	80 ppm	300 ppm	Genes
1	Cellular Regulation and Genomic Stability	0.906	0.984	1.666	*CDC25C*, *ERBB3*, *ITGB1*, *LAMB1*, *MSH6*, *MYC*, *NQO1*, *PPARG*, *PTEN*, *RB1*, *STAT3*, *TERT*, *TFRC*, *TGFBI*, *TIMP2*, *TOP1*, *XRCC5*
2	KRAS Effector and Hypoxic Response Module	0.792	0.929	1.565	*BCL2A1*, *CCNE1*, *CD44*, *EGFR*, *ETV6*, *FAT1*, *GADD45A*, *HIF1A*, *HSP90AB1*, *CXCL8*, *KMT2A*, *PGK1*, *PLAUR*, *RAD54L*, *RAF1*, *SFPQ*, *TPR*, *YY1*
3	TP53 Homeostatic Hub	0.982	0.975	1.460	*CAV1*, *CCND1*, *CEBPA*, *ERBB2*, *FOSL2*, *IGF1*, *JUN*, *MST1R*, *PCNA*, *PIM1*, *RARA*, *SIAH1*, *TP53*
4	Accessory Growth Signals (“Co-Conspirators”)	0.820	0.952	1.343	*BCL2*, *BCR*, *CDC25B*, *ETS1*, *ETS2*, *FAS*, *FGF2*, *IGFBP3*, *IRF1*, *LYN*, *MET*, *NRAS*, *PDGFA*, *PTPRG*, *SERPINE1*, *TYMS*, *XPC*
5	Invasion and Phenotypic Plasticity (STK11-Linked)	0.957	0.903	1.264	*BCL2L1*, *CCND3*, *CSK*, *HRAS*, *IGFBP2*, *NUMA1*, *S100A4*, *TGFB1*, *TYRO3*
6	Basal Maintenance and Survival Reserve	0.830	0.897	1.180	*AKT2*, *BIRC5*, *CDK2*, *CDK4*, *CDKN1A*, *CDKN2C*, *E2F1*, *ERCC2*, *FANCG*, *FGFR3*, *MTA1*, *MUC1*, *MYBL2*, *NPM1*, *CDK16*, *RET*, *TFE3*

Cluster: Numerical identifier for each cluster (1–6) as determined by GMM-6 clustering, ordered by the 300 ppm centroid coordinate. Function: Biological role associated with the cluster. 40 ppm, 80 ppm, 300 ppm: Centroid coordinates representing the average normalized gene expression in the cluster at each deuterium concentration, relative to the 150 ppm control. Genes: List of gene symbols assigned to the cluster. A more complete summary of the GMM-6 clusters, including detailed pathway and hallmark associations, is provided in Table S2.

### 3.7. Conclusions

Our study identifies deuterium concentration as a distinct modulator of the oncogenic expression in A549 lung adenocarcinoma cells. We observe that deuterium enrichment amplifies gene expression signatures linked to inflammation and invasion, whereas moderate depletion correlates with the systematic downregulation of key survival networks. Specifically, the suppression of *ABCB1*, *FGFR4*, and *MMP9* under depletion uncovers a potential transcriptomic vulnerability in the multidrug resistance and invasive machinery driven by the *KRAS/STK11/KEAP1* genotype. By resolving these dynamics through GMM-6 clustering and DBSCAN outlier detection, we define an expression window where metabolic constraints appear to counteract constitutive oncogenic drivers. Consequently, these data support a model in which deuterium depletion functions not merely as a general stressor, but as a candidate metabolic intervention, offering a transcriptomic rationale for re-sensitizing aggressive, drug-resistant tumors.

At the same time, several limitations constrain the scope of these conclusions. The analysis is based on a single LUAD model (A549) with a specific genotype, and the findings may not generalize to other molecular subtypes such as *EGFR*-driven, *ALK*-rearranged, or *KEAP1*-wildtype tumors. Furthermore, this study is based purely on gene expression data; the inferred biological outcomes regarding proliferation, drug response, and invasion require direct functional validation. Moreover, the in vitro conditions used here do not capture the metabolic and microenvironmental constraints present in vivo.

To address these limitations and extend these insights, systematic evaluation across additional LUAD cell lines with diverse mutational constellations is warranted, together with comparative studies in other malignancies such as prostate and ovarian cancers. Parallel profiling of healthy epithelial and stromal cells will be critical for distinguishing tumor-specific vulnerabilities from general metabolic responses. Expanding the range and increasing the number of tested deuterium concentrations will help define threshold effects, dose–response relationships, and context-dependent metabolic rewiring. Finally, integrating transcriptomic datasets with proteomic analyses will provide a deeper mechanistic understanding of how deuterium concentration reshapes signaling networks—potentially by modulating the metabolic substrates required for non-mutational epigenetic reprogramming—thereby refining its potential as a therapeutic axis in oncology.

## 4. Materials and Methods

### 4.1. Data Provenance and Biological Relevance

The gene expression data of the A549 lung cancer cells cultured in water media with four different deuterium concentrations: 40 ppm, 80 ppm, 150 ppm and 300 ppm were obtained from the authors of [9]. The dataset comprised expression measurements for 236 cancer-related genes (4 genes were measured twice, leading to 240 records), with each gene measured in technical duplicate at each of the four deuterium concentrations.

These four deuterium concentrations were selected to span the mechanistically relevant boundaries of deuterium modulation, representing the minimal set of points required to resolve the adaptive landscape. The 150 ppm control corresponds to the natural isotopic abundance of human body water, slightly smaller than the Vienna Standard Mean Ocean Water reference (VSMOW ≈ 155.76 ppm) and provides the appropriate physiological baseline for comparison. The 300 ppm enrichment level was selected based on prior toxicological and metabolic studies showing that moderate deuterium overload stimulates proliferation, increases oxidative signaling, and activates oncogenic transcription without inducing acute cytotoxicity, thereby modeling a hyper-oncogenic state. The 80 ppm condition represents the empirically validated therapeutic window: previous A549 studies and clinical deuterium-depleted water trials consistently identify 80–100 ppm as the range that maximally suppresses proliferation, reduces ROS-dependent signaling, and remains achievable in vivo. Finally, the 40 ppm deep-depletion condition was included to define the lower threshold of cellular adaptation. This extreme depletion induces a characteristic rebound activation of stress-response genes such as *ABCB1*, demonstrating that deuterium effects are non-linear and that excessive depletion triggers compensatory survival signaling. Together, these four concentrations delineate the adaptive landscape of deuterium-dependent transcriptional responses and are consistent with findings from in vivo studies [5,6,7,8] and human retrospective studies [15].

### 4.2. Filtering Logic and Reproducibility

To ensure data quality, we applied a multi-step filtering process to the raw gene expression dataset. First, variability between technical duplicates was quantified using a dimensionless coefficient of variation (CV = (x_1_ − x_2_)/(x_1_ + x_2_)), which provides a biologically interpretable measure of dispersion under low-replicate conditions. We then calculated propagated errors for expression ratios at 40, 80, and 300 ppm deuterium, and summed these values to obtain a total relative error for each gene. Genes with a summed error > 0.55 or an average copy number < 20 at the standard 150 ppm concentration were excluded. This approach effectively removed noisy, low-abundance measurements while retaining a high-confidence set of genes for downstream analysis. The distribution of summed relative propagated errors across all 236 genes is shown in Figure S4, while the subset of 110 genes retained after filtering is highlighted in Figure S5. The filtering algorithm was implemented in Python 3.13.12 and is publicly accessible through the A549-NanoString-analysis v1 GitHub repository, which also contains the input datasets and processed output files (https://github.com/csonkagi/A549-nanostring-analysis, accessed 3 March 2026).

### 4.3. Consensus Gene Curation

Next, we focused the analysis on a biologically relevant subset by selecting consensus cancer genes from the Cancer-Compass database (Consensus Cancer Genes, 15 Databases) [16]. This filtering step reduced the working gene set from 110 good-quality expression genes to 102 consensus cancer genes. The eight excluded genes are detailed in Table S3, together with their primary pathway associations and rationale for removal. Although these genes (e.g., *CTGF*, *GAPDH*, *GRB7*, *LIF*) are biologically important modifiers of tumor behavior, they lack recurrent driver mutations and multi-database support. Their exclusion strengthens the rigor of the 102-gene consensus list by ensuring that it reflects driver-level evidence rather than context-dependent or housekeeping roles.

### 4.4. Classification

Each gene was mapped to its canonical signaling pathway and annotated with relevant cancer hallmarks following the updated framework of Hanahan [17], which expanded the original hallmarks to include Nonmutational Epigenetic Reprogramming and Senescent Cells in the tumor microenvironment. This integrative approach ensured consistency between gene function, pathway assignment, and hallmark association. We build on recent reviews that emphasize the interplay of oncogenes and tumor suppressors [18], the role of epigenetic dynamics in tumor progression and therapy resistance [19], and the broader genetic landscape of cancer, including ligands, receptors, and signaling networks [20]. Genes were classified into four categories—oncogenes (driver mutations such as *HRAS*, *NRAS*, *MYC*, *BCL2*), tumor suppressors (*TP53*), tumor promoters (contextual modulators such as *TGFB1*, *PTGS2*, *SERPINE1*), and context-dependent regulators—based on curated literature evidence and mechanistic function [18]. This classification ensures a clear distinction between autonomous genetic drivers and microenvironmental enhancers.

### 4.5. Clustering Methodology

Then we sought to isolate genes exhibiting distinct expressions using the Density-Based Spatial Clustering of Applications with Noise (DBSCAN) algorithm [21]. This identified a unique set of 11 outlier genes. While these genes are of interest for their peculiar behavior, they were excluded from the next step to improve the stability and coherence of the Gaussian Mixture Model (GMM) [22] clustering on the remaining genes. The optimal number of GMM clusters was determined to be two, four or six based on silhouette scores (0.43–0.33, cf. Figure S3) [23]. For comparison, an alternative *k*-means clustering yielded a mean silhouette score of 0.26, indicating weak separation and substantial overlap between clusters. In contrast, GMM clustering provided higher assignment confidence and biologically coherent modules, supporting its use as the primary method.

To evaluate the sensitivity of Gaussian Mixture Model (GMM) clustering to individual gene removal, we performed a leave-one-out analysis using the Adjusted Rand Index (ARI) [24]. For each gene, we computed the ARI between the baseline clustering and the clustering obtained after excluding that gene. The ARI quantifies the similarity between two partitions, correcting for chance agreement, and ranges from −1 (complete disagreement) to 1 (perfect agreement). A higher ARI indicates greater clustering stability. The difference in ARI scores was used to identify genes that disproportionately influence clustering structure depending on model granularity.

To assess the confidence of gene-level cluster assignments, we computed the Mahalanobis distance of each gene from its assigned GMM cluster centroid. Mahalanobis distance accounts for the covariance structure of the data, providing a scale-invariant measure of how far a point lies from the center of a multivariate distribution. Unlike Euclidean distance, it penalizes directions of low variance more heavily, making it well-suited for evaluating membership strength in Gaussian-based models. Genes with high Mahalanobis distances are considered peripheral or borderline associated with their assigned cluster. In our analysis, Mahalanobis distance was used alongside ARI-based sensitivity scores to identify high-leverage genes and assess model robustness.

## Figures and Tables

**Table 1:** Centroid coordinates of pathway clusters in A549 lung adenocarcinoma cells cultured for 72 h under graded deuterium concentrations.

Pathway	40 ppm	80 ppm	300 ppm	Example Genes
Cytokine/inflammation	0.908	0.955	1.567	*IL6*, *CXCL8*, *PTGS2*
Cell survival	0.874	0.936	1.476	*TP53*, *BCL2*, *BIRC5*
Invasion & ECM remodeling	0.847	0.923	1.469	*CD44*, *MMP9*, *MUC1*
PI3K–AKT axis	0.880	0.971	1.468	*AKT2*, *PIM1*, *IGF1*
Transcriptional amplifiers	0.868	0.931	1.442	*MYC*, *MYCN*, *STAT3*
RAS–MAPK core	0.842	0.954	1.440	*HRAS*, *NRAS*, *RAF1*
**Global Center**	**0.884**	**0.940**	**1.436**	
DNA repair & stress tolerance	0.842	0.940	1.402	*ERCC2*, *MSH6*, *XPC*
RTKs & adaptors	0.959	0.948	1.377	*EGFR*, *ERBB2*, *FGFR1*
Cell cycle & replication	0.885	0.940	1.371	*CCND1*, *TERT*, *CDK4*
Drug Metabolism and Transport	1.122	0.585	1.268	*ABCB1*

Centroid values represent the mean relative expression ratios at 40, 80, and 300 ppm compared to the 150 ppm control. The Global Center corresponds to the overall average across all pathways and serves as a reference baseline. Pathways are annotated with representative genes.

## Data Availability

All gene expression data used in this study were obtained from previously published sources.

## Supplementary Materials

The following supporting information can be downloaded at: https://www.mdpi.com/article/10.3390/ijms27062605/s1.
