# Supplementary material for: Deuterium Concentration as a Dual Regulator: Depletion and Enrichment Elicit Divergent Transcriptional Responses in A549 Lung Adenocarcinoma Cells
Source: Int J Mol Sci. 2026 Mar 12;27(6):2605. doi: 10.3390/ijms27062605 (PMC13027021; doi:10.3390/ijms27062605)
Supplement: Supplementary file 1 [file ijms-27-02605-s001.zip › supp_ijms-4072276-new2CG.pdf]

Deuterium Concentration as a Dual Regulator: Depletion and Enrichment Elicit Divergent Transcriptional Responses in A549 Lung Adenocarcinoma Cells

Gabor I. Csonka [1,2], Ildikó Somlyai [3] and Gábor Somlyai [3]

[1] Department of Physics and Engineering Physics, Tulane University New Orleans, LA 70118; csonkagi@gmail.com

[2] Faculty of Chemical Technology and Biotechnology, Budapest University of Technology and Economics, 1111 Budapest, Műegyetem rakpart 3

[3] HYD LLC for Cancer Research and Drug Development, Budapest, Hungary; isomlyai@hyd.hu, gsomlyai@hyd.hu

Correspondence: csonkagi@gmail.com, Gábor I. Csonka (0000-0001-5701-4401) - ORCID,

Supporting information

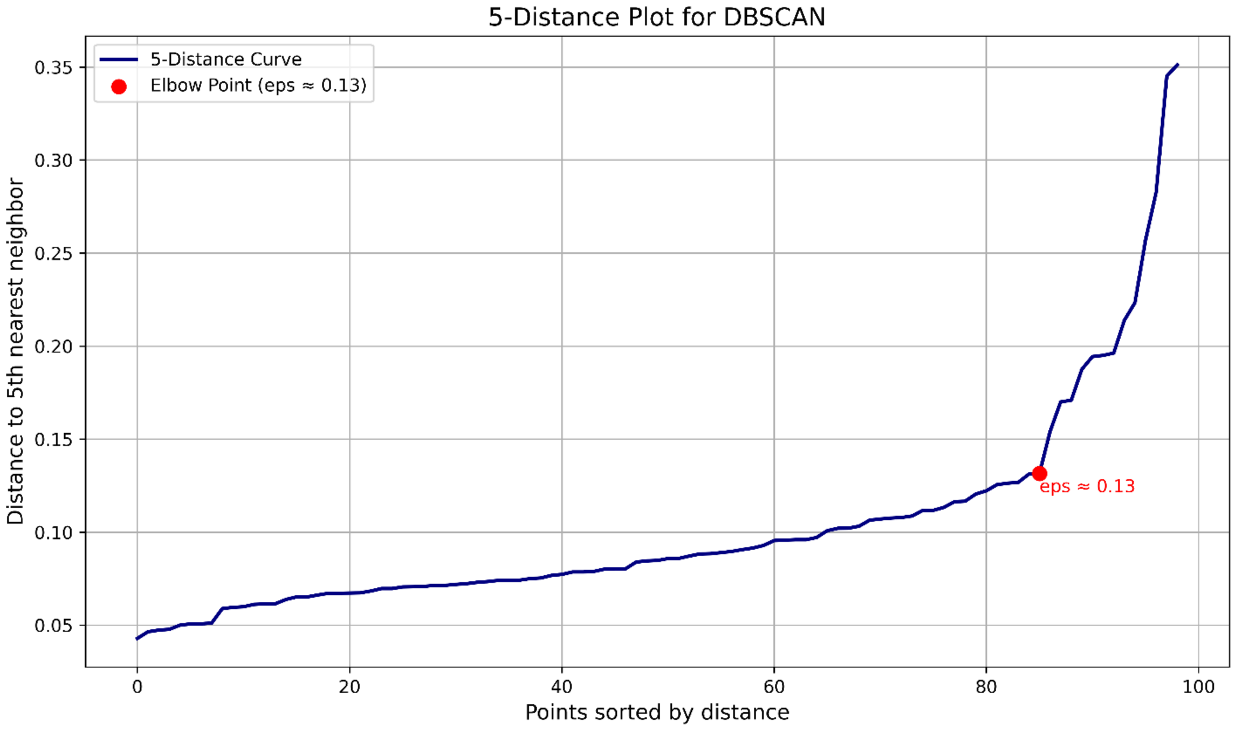

Fig. S1. 5-distance plot used for DBSCAN parameter selection.

The plot shows the sorted distances to the 5th nearest neighbor for all genes in the 3D expression space (ratios at 40, 80, and 300 ppm deuterium). The point of maximum curvature ("elbow") was used to select the ε parameter, following the standard practice of setting *min_samples* = 5.

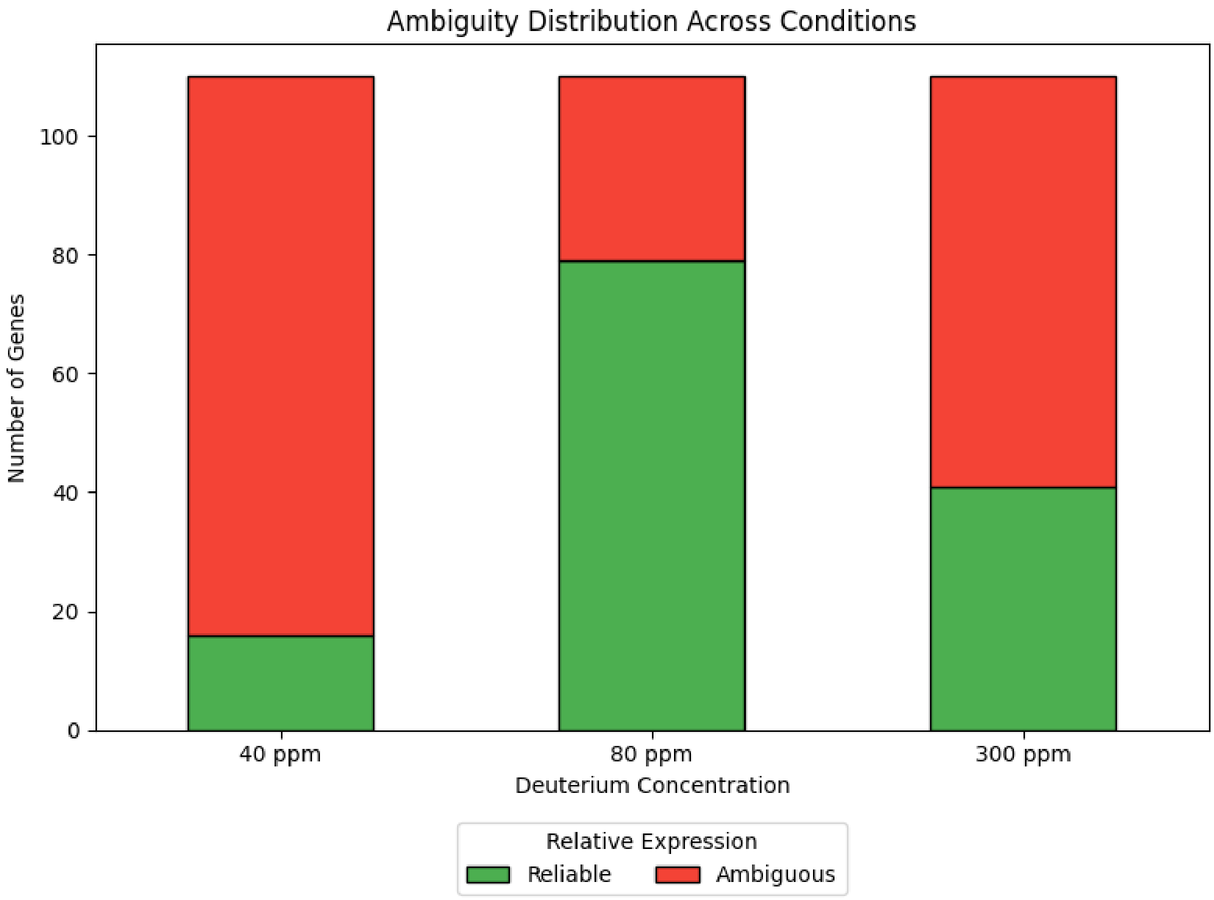

Fig. S2. Boundary ambiguity in symbolic pattern classification.
Scatter plot of relative expression ratios at 40, 80, and 300 ppm deuterium highlighting genes near symbolic classification thresholds. Genes with propagated measurement error in the 10–15% range are shown overlapping adjacent categories, illustrating how the five-symbol scheme can obscure differences between moderate and extreme expression changes (e.g., 40% vs. 100% overexpression both labeled ↑). These boundary effects motivate the use of complementary unsupervised clustering approaches such as Gaussian Mixture Models (GMM) to capture continuous variation and resolve ambiguous assignments.

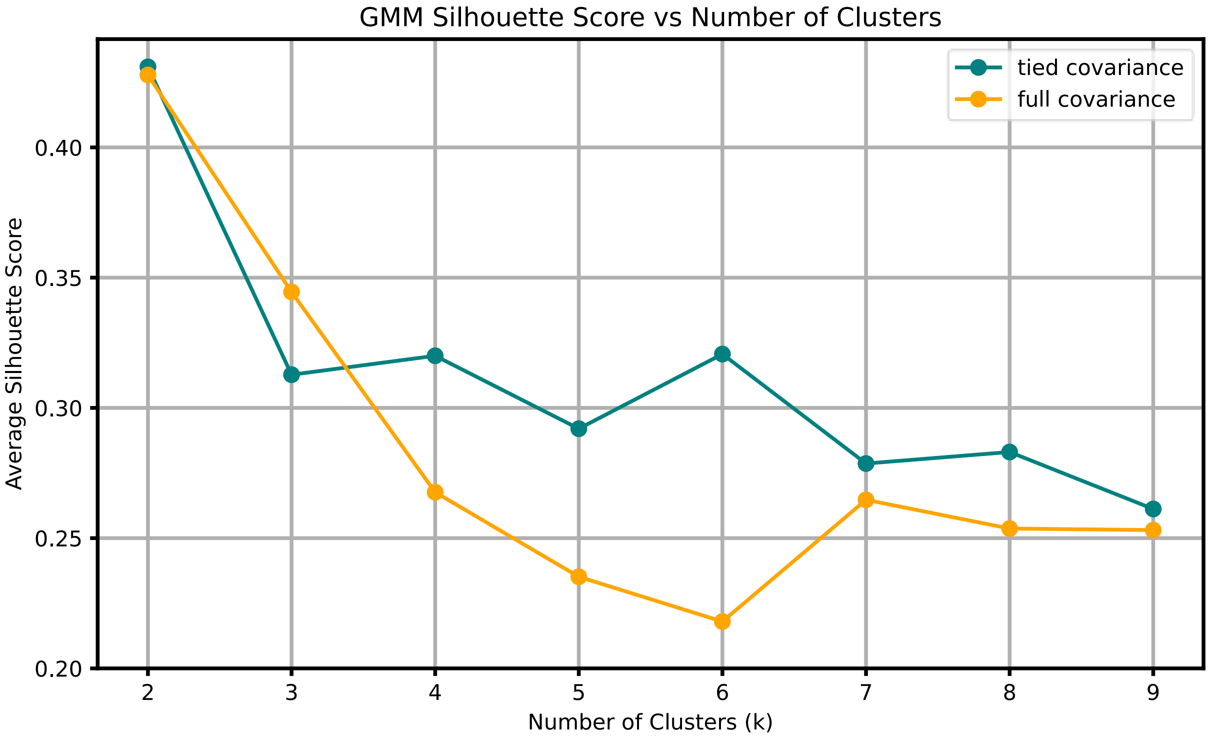

Fig. S3. Silhouette analysis of Gaussian Mixture Models (GMMs) across candidate cluster counts and covariance assumptions.

Average silhouette scores are shown for seven tested values of *k* (2–9), where *k* denotes the number of mixture components. Results are presented for both tied covariance (all clusters share a common covariance structure) and full covariance (each cluster has its own covariance matrix). Higher silhouette values indicate better separation and cohesion of clusters. This comparison was used to guide selection of the optimal cluster count and covariance model for capturing transcriptional variation under 40, 80, and 300 ppm deuterium conditions.

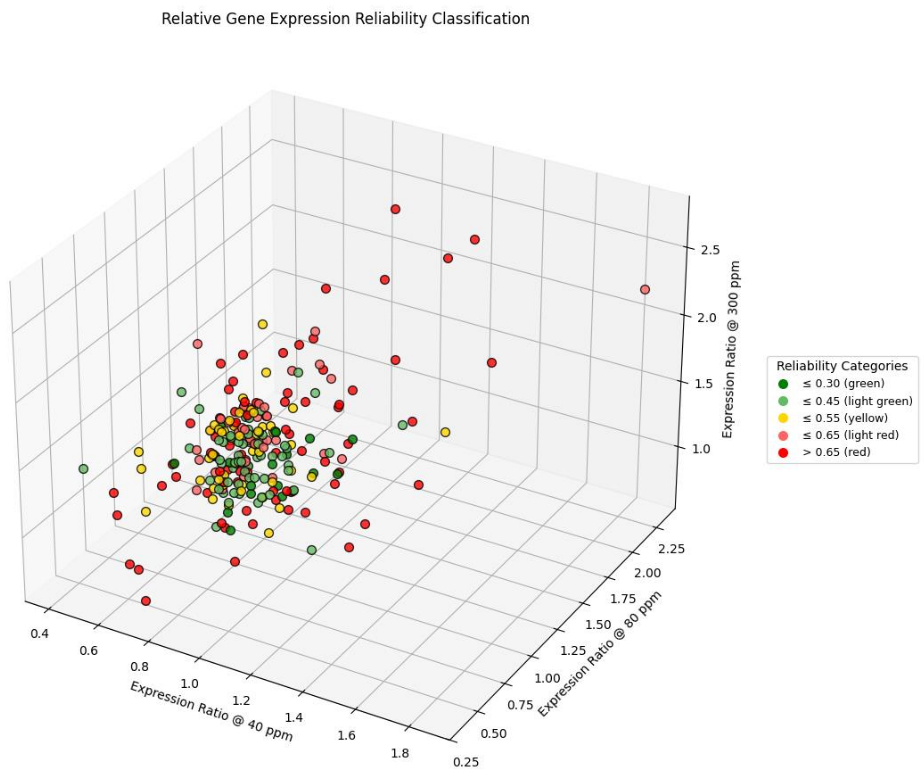

Fig. S4. Relative gene expression reliability classification (all genes).
Three-dimensional scatter plot of summed relative propagated errors for the full set of 236 cancer-related genes measured across 40, 80, and 300 ppm deuterium concentrations. Each point is color-coded by reliability class: ≤ 0.30 (green), ≤ 0.45 (light green), ≤ 0.55 (yellow), ≤ 0.65 (light red), and > 0.65 (red). Axes represent expression ratios at the three concentrations, illustrating the distribution of measurement precision across the dataset.

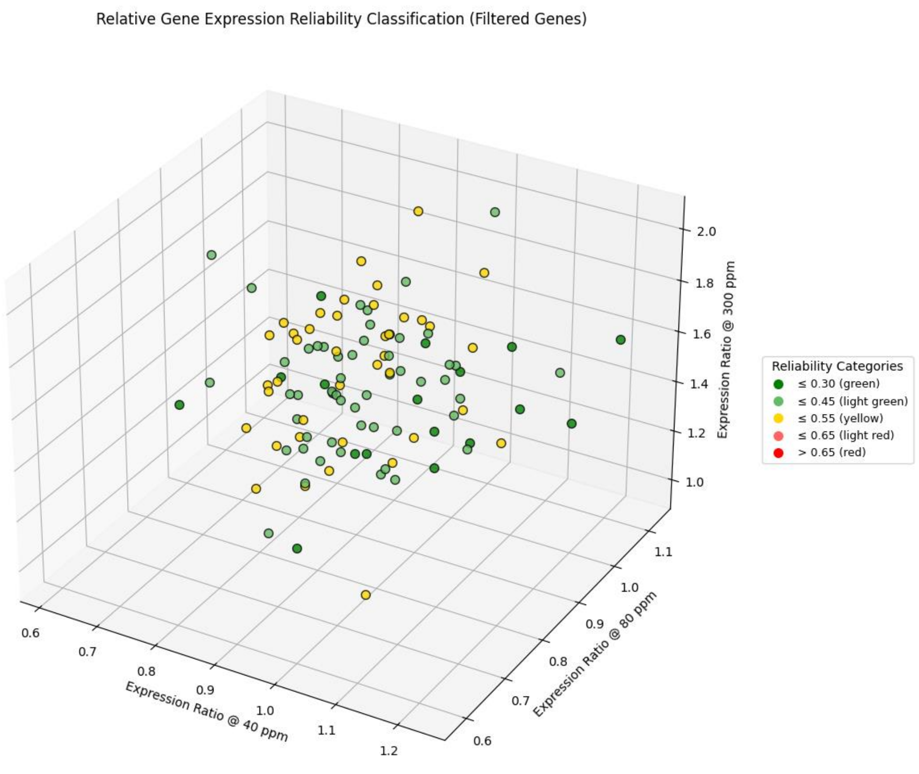

Fig. S5. Relative gene expression reliability classification (filtered genes). Three-dimensional scatter plot of the 110 genes retained after error-propagation filtering (total propagated relative error < 0.55). The same color scheme is applied as in Fig. S1. This figure highlights the subset of genes with reproducible, low-error measurements that form the basis for downstream clustering and pathway analysis.

Table S1. Canonical specific pathway-oriented knowledge edges

Edge (Pathway → Pathway)	Biological Rationale
**RTKs & adaptors → RAS–MAPK core**	Activated RTKs recruit adaptor proteins (e.g., GRB2/SOS) that catalyze GDP–GTP exchange on RAS, initiating the RAF–MEK–ERK cascade.
**RTKs & adaptors → PI3K–AKT axis**	RTKs directly activate PI3K via p85/p110 subunits, generating PIP3 and recruiting AKT to the membrane for phosphorylation.
**RAS–MAPK core → Cell cycle & replication**	ERK signaling induces transcription of cyclins (e.g., CCND1) and E2F targets, driving G1–S transition.

Edge (Pathway → Pathway)	Biological Rationale
**RAS–MAPK core → Transcriptional amplifiers**	MAPK/ERK phosphorylates transcription factors (e.g., *MYC, JUN, ETS*), amplifying proliferative gene expression.
**PI3K–AKT axis → Apoptosis evasion**	AKT phosphorylates and inactivates pro-apoptotic proteins (BAD, caspase-9) and stabilizes anti-apoptotic *BCL2* family members.
**PI3K–AKT axis → Cell cycle & replication**	AKT promotes G1–S transition by upregulating cyclin D1 and inhibiting p21/p27, coordinating growth and metabolism.
**Cytokine/inflammation → Transcriptional amplifiers**	Cytokines (e.g., IL6, TNFα) activate STAT3, NF-κB, and HIF1A, which function as transcriptional amplifiers of oncogenic programs.
**Cytokine/inflammation → Invasion & ECM remodeling**	Inflammatory cytokines (IL6, TGF-β) induce EMT and upregulate MMPs, linking inflammation to invasion and metastasis.

Table S2. GMM-6 Cluster: Cluster ID, Function Centroid Coordinates, Pathways, Hallmarks, Gene Count; NanoString Code Set: List of Genes and GMM-4 and GMM6-6 Cluster Stability Data (see attached Excel File)

- All gene codes used in the NanoString panel
- Cluster assignments, Functional annotations (pathways, hallmarks)
- Expression stability and error metrics across conditions

Table S3. Genes excluded from the cancer gene set

Eight genes were removed during filtering against the Cancer Compass Consensus Cancer Genes (15 Databases) list. Although biologically relevant as modifiers of tumor behavior, they lack recurrent driver mutations and multi-database support. Their exclusion ensures that the final 102-gene set reflects driver-level evidence only.

Gene	Main Pathway(s)	Rationale for Exclusion
CTGF	TGF-β signaling, PI3K–AKT, ECM remodeling	Context-dependent effector of invasion/fibrosis; not annotated as a driver gene across databases
GAPDH	Glycolysis, metabolic reprogramming, oxidative stress	Housekeeping enzyme with moonlighting roles; not a recurrently mutated cancer driver
GRB7	RTK signaling (HER2/EGFR), PI3K–AKT, MAPK	Adaptor protein amplifying oncogenic signaling; lacks consistent driver mutation evidence
GUSB		Housekeeping enzyme; no recurrent driver role.
HMMR	Cell cycle, spindle assembly, ECM–receptor interaction	Overexpressed in cancer, but not a consensus driver gene
LIF	JAK–STAT3 signaling, cytokine signaling	Promotes stemness and immune modulation; excluded due to lack of recurrent driver mutations
OGG1	Base excision repair, oxidative stress response	DNA repair enzyme; polymorphisms linked to risk, but not a canonical driver
TUBB	Cytoskeleton organization, mitotic spindle, drug response	Structural protein and chemotherapy target; not a recurrently mutated driver
